# A mixed methods evaluation of Advanced Life Support in Obstetrics (ALSO) and Basic Life Support in Obstetrics (BLSO) in a resource-limited setting on the Thailand-Myanmar border

**DOI:** 10.12688/wellcomeopenres.16599.2

**Published:** 2021-06-28

**Authors:** Rose McGready, Marcus J. Rijken, Claudia Turner, Hla Hla Than, Nay Win Tun, Aung Myat Min, Sophia Hla, Nan San Wai, Kieran Proux, Thaw Htway Min, Mary Ellen Gilder, Anne Sneddon

**Affiliations:** 1 Shoklo Malaria Research Unit, , Mahidol-Oxford Tropical Medicine Research Unit, Faculty of Tropical Medicine, Mahidol University, Mae Sot, Tak, 63110, Thailand; 2 Centre for Tropical Medicine and Global Health, Nuffield Department of Medicine, University of Oxford, Old Road Campus, Oxford, OX3 7LG, UK; 3 Utrecht University Medical Centre, Utrecht University, Utrecht, Utrecht, 3584 CX, The Netherlands; 4 Julius Centre Global Health, University Medical Center, Utrecht, Utrecht, 3508 GA, The Netherlands; 5 Cambodia-Oxford Medical Research Unit, Angkor Hospital for Children, Siem Reap, Cambodia; 6 Reproductive Health, Mae Tao Clinic, Mae Sot, 63110, Thailand; 7 Department of Family Medicine, Faculty of Medicine, Chiang Mai University,, Chiang Mai, Chiang Mai, 50200, Thailand; 8 School of Medicine, Gold Coast campus, Griffith University, Queensland, 4222, Australia

**Keywords:** Adult education, Kirkpatrick’s framework, post-partum haemorrhage, maternal mortality, stillbirth, self-confidence, advanced life support in obstetrics training, post-conflict, resource-limited setting

## Abstract

**Background: **Short emergency obstetric care (EmOC) courses have demonstrated improved provider confidence, knowledge and skills but impact on indicators such as maternal mortality and stillbirth is less substantial. This manuscript evaluates Advanced Life Support in Obstetrics (ALSO) and Basic Life Support (BLSO) as an adult education tool, in a protracted, post-conflict and resource-limited setting.

**Methods: **A mixed methods evaluation was used. Basic characteristics of ALSO and BLSO participants and their course results were summarized. Kirkpatrick’s framework for assessment of education effectiveness included: qualitative data on participants’ reactions to training (level 1); and quantitative health indicator data on change in the availability and quality of EmOC and in maternal and/or neonatal health outcomes (level 4), by evaluation of the post-partum haemorrhage (PPH) related maternal mortality ratio (MMR) and stillbirth rate in the eight years prior and following implementation of ALSO and BLSO.

**Results: **561 Thailand-Myanmar border health workers participated in ALSO (n=355) and BLSO (n=206) courses 2008-2020. Pass rates on skills exceeded 90% for both courses while 50% passed the written ALSO test. Perceived confidence significantly improved for all items assessed. In the eight-year block preceding the implementation of ALSO and BLSO (2000-07) the PPH related MMR per 100,000 live births was 57.0 (95%CI 30.06-108.3)(9/15797) compared to 25.4 (95%CI 11.6-55.4)(6/23620) eight years following (2009-16), p=0.109. After adjustment, PPH related maternal mortality was associated with birth before ALSO/BLSO implementation aOR 3.825 (95%CI 1.1233-11.870), migrant (not refugee) status aOR 3.814 (95%CI 1.241-11.718) and attending ≤four antenatal consultations aOR 3.648 (95%CI 1.189-11.191). Stillbirth rate per 1,000 total births was 18.2 (95%CI 16.2-20.4)(291/16016) before the courses, and 11.1 (95%CI 9.8-12.5)(264/23884) after, p=0.038. Birth before ALSO/ BLSO implementation was associated with stillbirth aoR 1.235 (95%CI 1.018-1.500).

**Conclusions: **This evaluation suggests ALSO and BLSO are sustainable, beneficial, EmOC trainings for adult education in protracted, post-conflict, resource-limited settings.

## Introduction

Skilled attendance at birth providing quality emergency obstetric and newborn care is an essential element for reducing the high burden of maternal and neonatal morbidity and mortality in resource-limited settings (RLS) ^1, 2^.

A number of practical skills or “hands-on” short course courses are aimed at improving emergency obstetric care (EmOC) ^3^. The Advanced Life Support in Obstetrics (ALSO) course developed by the American Academy of Family Physicians is one of the oldest commencing in 1991, is standardized and evidenced-based, and available in multiple languages ^4, 5^. ALSO promotes inter-professional and multidisciplinary training to equip the maternity team (nurse midwives, registered nurses, physicians, residents and other members) to improve patient safety and positively impact maternal and newborn outcomes in obstetric emergencies. Positive ALSO evaluations from high-income countries ^6, 7^ and low-income and RLS (Colombia, Guatemala, Honduras, and Tanzania) report decreased in‐hospital maternal mortality, episiotomy use, and post-partum haemorrhage (PPH); and active management of third stage of labour and vacuum‐assisted vaginal delivery increased in frequency after ALSO training ^8^. In 2010 a systematic review in low-resource environments suggested there was a lack of strong evidence of the effectiveness of emergency obstetric trainings ^9^. In 2020 a global review reported improved provider knowledge/skills and change in clinical practice but evidence for a reduction in the number of cases of post-partum haemorrhage (PPH), case fatality rates, stillbirths and institutional maternal mortality were less strong ^3^.

The ALSO approach to learning is adult based. Learning suits professionals working in government health systems where tertiary education and certification of health care training is the norm. Tertiary qualified staff may not be available in post-conflict and politically unstable RLS with broken education and health infrastructure ^10^. Evidence for the type of learning and teaching suitable for healthcare workers in politically unstable and post-conflict settings is limited ^11^. ALSO requires a degree of health literacy that may challenge healthcare workers based in countries rebuilding education and health systems ^12, 13^.

Basic Life Support in Obstetrics (BLSO) is complementary to ALSO but structured for pre-hospital health care providers, first responders, and medical, nursing and physician assistant students ^14^. The objective of BLSO is to improve the management of normal deliveries, as well as obstetric emergencies by standardizing the skills of frontline responders. BLSO is more difficult to compare internationally as there is more flexibility on topics included in the course, realizing the need for context appropriate training.

Reported success of training in emergency obstetric care in low-income countries within government health systems is commendable but are these replicable in post-conflict or politically unstable health care systems? ^10, 15– 18^ Shoklo Malaria Research Unit (SMRU), a humanitarian and research organization on the Thailand Myanmar border, has provided antenatal care and childbirth facilities for refugees since 1986 and migrants since 1998. Facility based ALSO and BLSO training for Karen and Burmese healthcare workers was introduced in 2008. This was the first time the ALSO course has been held in a refugee camp. The objective of this analysis is to evaluate ALSO and BLSO as an adult education tool in emergency obstetric care, from the first course in 2008 to the most recently completed course in May 2020, in a protracted, post-conflict, RLS.

## Methods

### Study design

This mixed methods study collates several data sources to evaluate ALSO and BLSO as an adult education tool over the time period of 2008 to 2020. Before each course participants provided information about themselves using a standard form. Change in perceived confidence was measured before and after the course, and a sub-cohort (of the 2008 course) had a 12-month evaluation. The course theory and skill results were summarized. Course strengths were evaluated through an open-ended question. Finally, obstetric indicators including PPH related maternal mortality and stillbirth in the eight year block preceding the implementation of ALSO and BLSO and in eight years after were sourced from routine surveillance data.

### Setting

**Figure 1.**
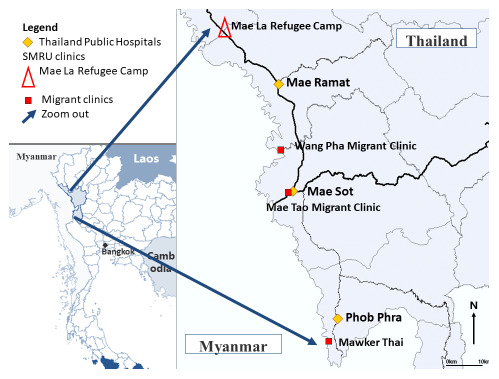
Map of the area and location of clinics where ALSO and BLSO were conducted.

***Maternal and child health care.*** Refugees from Myanmar have lived in camps on the Thailand side of the border since 1984 with primary health care provided by a coalition of non-government organizations (NGO). Shoklo Malaria Research Unit (SMRU) was established in Shoklo Refugee Camp in 1986 conducting research and providing humanitarian care. SMRU commenced maternal health operations outside the camps with border migrant populations in 1998. Mae Tao Clinic (MTC) established in 1988 provides humanitarian care to Karen and Burmese in Mae Sot. SMRU and MTC clinics provide similar maternal and child health services including antenatal care (ANC), birthing services, post-natal care and special care baby units. SMRU, MTC and NGO services do not have capacity for advanced interventions including caesarean sections. If caesarean section is indicated women are transferred to the nearest Thai Public Hospital, 30–90 minutes away depending on the clinic ( Figure 1).

Initially, *P. falciparum* infection accounted for the largest proportion of maternal mortality (estimated at 1,000 per 100,000 live births in 1985–1986 ^19^) and was the focus of activity for the first two decades. In 1994 basic tools such as the World Health Organization (WHO) partogram in labour and essential medications such as magnesium sulphate for eclampsia and severe pre-eclampsia were introduced. To standardize interventions for common obstetric emergencies and medical complications SMRU produced a three language (Karen, Burmese and English) ‘Manual of Obstetrics’ in 1998, which has subsequently been revised three times (English only). As post-partum haemorrhage (PPH) ^20^ replaced malaria as the largest cause of maternal mortality, the need further training in obstetric emergencies was recognized.

### Participants

***Human resources.*** There are significant barriers to displaced persons in Thailand (refugees or migrants) obtaining formal training and certification as health workers in either Thailand or Myanmar. For more than three decades healthcare work by NGOs and humanitarian organizations on the border including SMRU and MTC have been provided by local Karen and Burmese from the community trained to work as medics, nurses, midwives, sonographers or counsellors as defined here:

*Medics*: A ‘medic’ takes the role of doctor by doing clinical examinations, ordering laboratory tests, making the diagnoses and prescribing treatment. They have normally received a minimum of 6–12 months of theory training and a further 6–12 months of on the job practical training ^12, 13^.

*Nurse*: A ‘nurse’ works in the same capacity as nurses in developed countries and have normally received 3–6 months of theory and 3–6 months of practical training.

*Midwife*: A ‘midwife’ (or skilled birth attendant) provides antenatal and delivery care services. SMRU developed its first curriculum in 1994, with the current course taking a minimum of 15 months (30 deliveries) ^21^.

*Sonographer*: SMRU trains local staff in ultrasound gestational age assessment. They have the hardest entrance test compared to the other health staff and the quality of their work is reported elsewhere ^22^.

*Counsellor*: A ‘counsellor’ provides antenatal care counselling particularly in explaining screening for pregnant women (blood tests, gender-based violence, mental health, nutrition).

*Myanmar doctors*: SMRU employs doctors to support the health workers and some come from Myanmar.

All these health workers were invited to participate on courses as they were offered.

### ALSO and BLSO

With support from ALSO Australasia, it was decided to launch the program at SMRU and MTC. The initial course was supported by four instructors from Australia. The ALSO course includes a syllabus to be studied in advance of the course, an intensive two-day course reviewing obstetric emergencies, and an examination of theory and skills ( Table 1). The BLSO course is conducted in one to two days, context adapted for front line responders ( Table 1), with the emphasis on skills.

**Table 1.** Situations taught in the ALSO and BLSO course on the Thailand-Myanmar Border.

ALSO	BLSO
Safety in Maternity Care	Safety in Maternity care/Universal precautions
	Normal labour
Intermittent auscultation (as opposed to Intrapartum fetal surveillance)	Emergent/Urgent prenatal assessment (no electronic fetal monitoring option)
Labour dystocia	
Forceps and Vacuum -assisted births	
Shoulder dystocia	Shoulder dystocia
Pre-eclampsia/Eclampsia	Pre-eclampsia/Eclampsia
Vaginal breech births/malpresentation	Breech, Twin gestation, Cord Prolapse and other malpresentation
Umbilical cord prolapse	
Preterm labour (PTL)	PTL and Preterm, premature rupture of membranes (PPROM)
Antepartum haemorrhage (APH)	Haemorrhage (1st trimester, APH and PPH)
Postpartum haemorrhage (PPH)	
First-trimester bleeding	
Maternal resuscitation	Maternal resuscitation
Maternal venous thrombosis ^^^	
Severe malaria and sepsis ^^^	
Neonatal resuscitation (NNR) ^^^^	Neonatal resuscitation (NNR)
Perinatal loss/Birth crisis	

^As maternal venous thrombosis mortality in the area is much lower than mortality from severe malaria and sepsis; it was de-emphasized while severe malaria and sepsis were included from 2008. ^^USA and Australasia deviate slightly on content in different editions of ALSO so the 9 ^th^ Edition USA does not include neonatal resuscitation but it is retained on the Thailand-Myanmar border as no alternative course is available.

***Instructors for ALSO and BLSO.*** ALSO and BLSO instructors must have successfully passed both ALSO and the ALSO Instructors course, detailed elsewhere but briefly a short intensive training of the ALSO teaching methods ^4, 5^. A range of languages are used in the clinics with most local staff speaking one or two dialects of Karen (Sgaw Karen or Pho Karen), Burmese and English as a 2 ^nd^, 3 ^rd^ or 4 ^th^ language. Following the first course, local staff were selected as Instructors based on qualities such as being a good team player, performing well on the actual ALSO course and languages spoken ^23^. Courses on the border retain the recommended participant to instructor ratio as 4:1.

***ALSO for the Thailand-Myanmar border.*** SMRU course organizers reduced the volume and complexity of the English course material to reflect the baseline education of the group and to ensure the content was relevant to the local setting e.g. including malaria and sepsis as a cause of maternal mortality ( Table 1). While the Thailand-Myanmar border commenced with ALSO, the Global ALSO manual has emerged as the most relevant tool to adapt the ALSO course to local circumstances, especially in resource limited settings ^24^. ALSO course teaching was structured into two parts: the preparation phase and the standard two day intensive course. The preparation phase reviewed one to two chapters per week, mostly in English (60–120 minutes, led by a doctor). A study group for 2–3 hours later in the week, mostly in Karen and Burmese language led by a senior midwife or medic included practice of skills using donated mannequins. The total preparation phase took approximately 12–16 weeks.

The ALSO written exam was also modified for relevance and clarity, removing difficult English e.g. double negatives, and intrapartum (electronic) fetal surveillance, leaving 50 questions. The exams were available in Burmese starting in 2008 and Karen in 2013. Translation verification included back translation. Courses and exams were updated regularly, and numerical scores were given to participants.

***BLSO for the Thailand-Myanmar border.*** BLSO was directed at frontline staff in border clinics who might encounter pregnant women or neonates in their work. English literacy was a recognized barrier to learning for these staff. Student midwives with little or no clinical experience were also encouraged to participate in BLSO before the end of the first year of training to promote confidence in the workplace and familiarity with some emergencies.

BLSO does not have a preparation phase for participants, the content and the assessment all took place in a two-day course. Material was prepared in English and taught in Burmese and Karen, with a 12-page handout (>50% pictures) provided.

The BLSO was assessed by 10 short answer questions and was available in four languages (Burmese, Karen, Thai and English). The practical component assessed emergencies that were likely to be seen in the community and scored as pass or fail.

***Course fees and size.*** Sustainability in high-income countries comes at a cost to the participant. In this RLS both ALSO and BLSO were conducted by SMRU without cost to the participant. Doctors were asked to pay for the cost of copying the materials. Course size was limited by mannequins to a maximum of eight per group and three groups i.e. 24 participants at a single course.

### Evaluation of adult education effectiveness

Kirkpatrick’s framework for assessment of effectiveness of adult education includes four levels of data: (level 1) participants’ reactions to training, (level 2) change in knowledge and/or skills, (level 3) change in behaviour or clinical practice and (level 4) change in the availability and quality of EmOC and in maternal and/or neonatal health outcomes ^25^. In this analysis data on level 1 and level 4 were measured.

All level 1 data was voluntary following a verbal explanation to participants at course orientation. Two measures were included: perceived confidence and self-reported strengths of the course. Prospective repeated-measures surveys at three time periods on perceived confidence: pre course, immediate post course and in a sub-cohort from the original ALSO course in 2008, at 12 months post baseline. The survey tool was adapted with permission from ALSO courses in the USA ^6, 26^.

Self-reported strengths of the course were measured for ALSO participants by response to an open-ended survey question: “What are the strengths of the course?”. Answers in native language were encouraged with responses in Karen and Burmese translated to English.

Level 4 data were extracted from the routine birth facility data curated by SMRU, and measured pre and post ALSO and BLSO in two equal year blocks: before (2000-2007) and after (2009-2016) implementation. The eight year time frame was chosen as all archived data were cleaned and data checked, while more recent data was not.

### Variables

Participant characteristics included age, gender, profession, years of practice, workplace, high school and university attendance, and theory and skills results (as a percentage).

For qualitative analysis perceived confidence to manage the obstetric emergency situations covered in the ALSO or BLSO curriculum (detailed in Table 1) was measured using a four-point [1 terrified, 2 scared, 3 coping, 4 comfortable] rank-ordered response Likert scale.

For quantitative analysis extracted variables related to maternal mortality and stillbirth included: status (migrant/refugee), parity, smoking, underweight (<40 kg), four or less antenatal consultations, anaemia the first antenatal visit (haematocrit < 30%) and falciparum malaria confirmed by microscopy by active and frequent screening in pregnancy.

### Statistical analysis

The analysis was conducted using SPSS 23.0 for Windows. Participant characteristics such as gender were summarized mostly by proportions (n, %) while age and scores on the course were summarized using the median (interquartile range). The Likert scale responses were summarized by median, interquartile range (IQR) or range (minimum-maximum (min-max)), or proportions, as appropriate. The 95% confidence intervals of proportions were calculated using Wilson’s method ^27^. The comparison of confidence scores was based on individual paired testing using the Wilcoxon signed rank test.

Qualitative data were analyzed using conventional content analysis to identify major themes ^28^. Two authors independently and manually reviewed printed transcripts of the data and suggested possible codes. After discussion, and analysis of the relationship between proposed codes, three common themes were agreed on for the final analysis. Data review was repeated and content was summarized into these three themes. Each reviewer independently chose exemplary comments for each of the three themes and final inclusion was based on discussion and by mutual agreement.

A word cloud was developed using free online software Voyant Tools ( https://voyant-tools.org/), to examine the most commonly used words from all the responses to the open-ended question.

The odds ratio (OR) and confidence interval (CI) for factors associated with the binary outcomes, PPH related maternal mortality (yes/no) and stillbirth (yes/no), were calculated by univariate analysis. The outcome of interest, birth before ALSO and BLSO implementation (yes/no) was retained in the models, as were factors with a p < 0.10 in univariate analysis. These factors were entered in their respective logistic regression model, and were included in the relevant tables, along with adjusted odds ratios (aOR) and 95% CI.

### Bias

The participants include all those who participated to the ALSO and BLSO courses reducing selection bias. The qualitative data was analyzed by two of the local instructors who support the course and therefore are at risk of ‘positive’ bias however they are not responsible for the numerous volunteered responses from the participants. The quantitative data is limited to what data was available and at risk of not being unable to account for factors that can affect maternal mortality and stillbirth. For example ‘socio-economic status’ was not available and may have contributed to improvements although refugees and migrants have remained marginalized populations over the entire period.

### Ethical approval

As part of standard procedure in this setting the details of this project were presented, discussed and approved by the local Community Advisory Board (TCAB-311-12,016), Mae Sot Thailand. No ethical approval was sought for the education evaluation data which was fully anonymized and previously collected. Oxford University Ethics Committee, ethical approval reference code OxTREC 28-09 was obtained for extraction of PPH related mortality and stillbirth data from anonymized hospital records.

## Results

### First ALSO in a refugee camp and migrant clinics

In 2008, ALSO training took place for the first time in a refugee camp with 18 participants from Maela Camp; and in Wang Pha clinic where 19 participants came from three border clinics (Wang Pha, Maw Ker Thai and Mae Tao Clinic in Mae Sot). The majority of participants were midwives 78.4% (n=29/37), 73.0% (n=27/38) had five or less years of work experience ^26^. Only 16.2% (n=6/37) had been to university in Myanmar and none had the opportunity to study courses related to medical health care. In total, 94.7% (n=35/37) passed the practical exam while only 29.7% (n=11/37) successfully passed the theory component to ALSO standards. Eleven participants were invited to become instructors to facilitate future courses in local languages.

### Subsequent ALSO and BLSO courses

**Figure 2.**
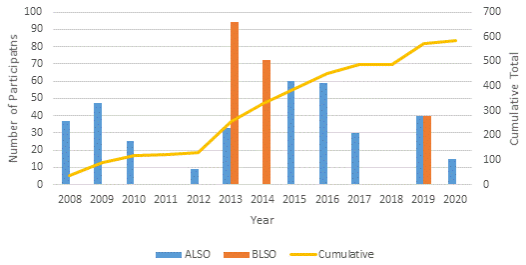
Time frame of the conduct of ALSO and BLSO courses on the Thailand-Myanmar border 2008-2020 including numbers of participants. ALSO=Advanced Life Support in Obstetrics; BLSO=Basic Life Support in Obstetrics.

From 2008 to 2020, 561 health workers participated in courses conducted at SMRU and MTC: 355 in ALSO and 206 in BLSO ( Figure 2).

Local participant characteristics included ( Table 2) young age (median <30 years), female predominance 84.8% (476/561) with a majority with five or less years of practice. There was a high uptake to the courses by midwives (none were male), and a variety of workplaces (all non-tertiary) were involved. Less than four years high school was reported by one in four participants and university attendance (not completion) in less than one in five participants

**Table 2.** Characteristics of participants on the ALSO and BLSO courses.

Characteristics		ALSO	BLSO
		n=355	n=206
Age	median [min-max]	29 [19–54]	25.5 [18–59]
Female		88.2 (313)	79.1 (163)
High school	less than 4 years	28.5 (101/355)	20.4 (41/201)
Myanmar University	attended (degree maybe incomplete)	18.3 (64/349)	14.9 (30/201)
First time on course		75.2 (267)	97.6 (201)
Health profession	medic	18.3 (65)	26.2 (54)
	nurse	13.5 (48)	23.3 (48)
	midwife	62.8 (223)	35.9 (74)
	doctor (Myanmar)	2.5 (9)	0
	Sonographer	0.8 (3)	4.9 (10)
	Counsellor	1.1 (4)	2.4 (5)
	Other e.g. pharmacist	0.3 (1)	4.9 (10)
	not recorded	0.8 (3)	2.4 (5)
Years in practice	≤1	20.0 (71)	63.1 (130)
	2–5	38.6 (137)	21.8 (45)
	6–10	25.9 (92)	8.7 (18)
	11–15	11.8 (42)	2.9 (6)
	≥16	3.4 (12)	1.0 (4)
	missing	0.3 (1)	3 (1.5)
Work place	Maela Refugee camp	33.2 (118)	57.3 (118)
	WPA and MKT migrant sites	32.1 (114)	22.3 (46)
	Mae Tao Clinic Mae Sot	15.5 (55)	0
	IRC Myanmar doctors	2.5 (9)	0
Theory results (%)	score median [min-max]	66 [11–96]	80 [35–100]
	passed 1st attempt	26.8 (95) *	93.2 (192) ^
	passed on retake	50.6 (43/85)	70.0 (7/10)
Skills results	score median [min-max]%	93 [35–100]	n.a.
	passed 1st attempt	98.6 (350)	91.7 (189)

*≥70%, ^≥60% *Data in % (n), or otherwise indicated, Missing data: ALSO age n=1, BLSO age n=3* *Abbreviations: ALSO=Advanced Life Support in Obstetrics; BLSO=Basic Life Support in Obstetrics, AMI Aide Médicale Internationale, IRC International Rescue Committee, MKT Maw Ker Thai, MRML Mae Ra Mu Wang, MTC Mae Tao Clinic, WPA Wang Pa*

The main written and spoken language of participants confirmed that no one language was available to all participants ( Table 3).

**Table 3.** Languages spoken and written by participants on the ALSO and BLSO courses.

	Language	ALSO	BLSO
**Spoken**	Poe Karen	15.2 (53/348)	11.2 (23/203)
	Sgaw Karen	91.7 (319/348)	92.1 (187/203)
	Burmese	98.0 (34/348)	98.0 (199/203)
	English	69.3 (241/348)	70.4 (143/203)
	Thai	13.5 (47/348)	18.2 (37/203)
	other	3, (Lisu, Mon, Shan)	1, (Karenni)
**Written**	Poe	10.3 (36/348)	8.4 (17/203)
	Sgaw	80.2 (279/348)	85.2 (173/203)
	Burmese	96.6 (336/348)	96.6 (196/203)
	English	87.1 (303/348)	78.6 (162/203)
	Thai	10.3 (36/348)	10.8 (22/203)
	other	1 (Lisu)	1 (Karenni)

*ALSO=Advanced Life Support in Obstetrics; BLSO=Basic Life Support in Obstetrics*. Missing data: n=7 ALSO, n=3 BLSO

### Results of the ALSO and BLSO courses

Results on the skills component of ALSO have been consistently high, with a median score of 93 [35-100], and 98.6% (n=350/355) passing on first attempt. This contrasts to the theory component with a median score 66 [11-96], with 26.8% (95/355) passing on the first attempt, and one in two participants passing on a retake (50.6%; n=43/85). Retakes were not offered in 2008.

Pass rates for the less demanding BLSO course were high for both the practical (91.7%; n=189/206) and the theory (93.2%; n=192/206).

Overall, 88.9% (8/9) doctors passed both practical and theory but all attended tertiary education.

### Changes in perceived confidence

**Figure 3.**
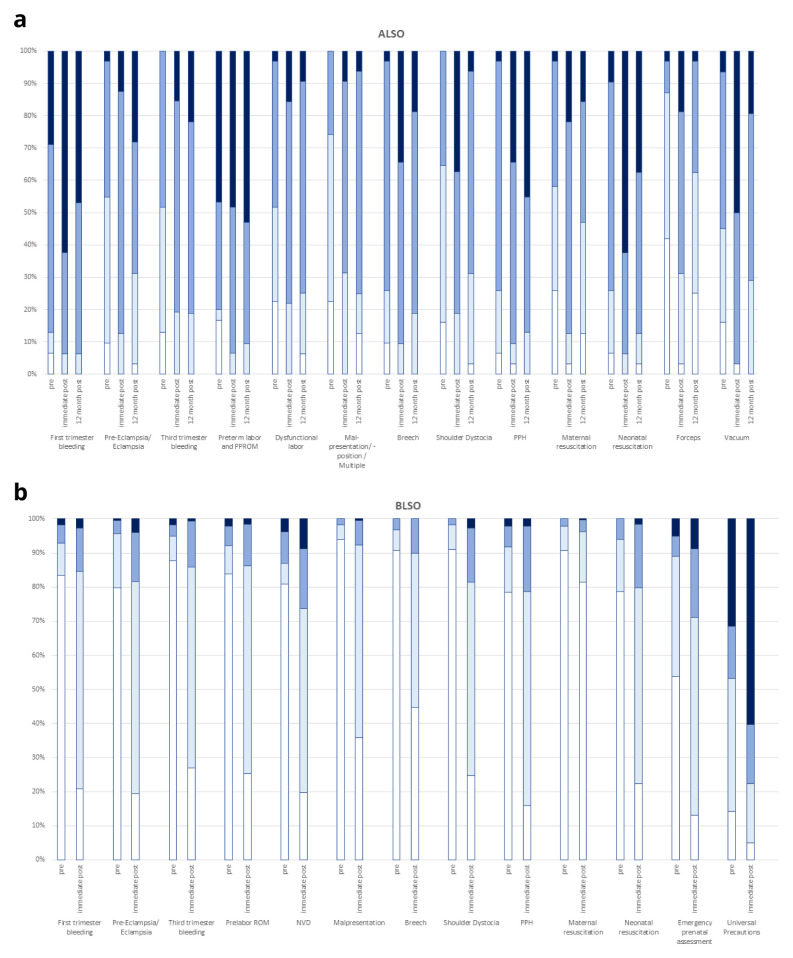
Evaluation of perceived confidence pre and post course evaluation. Panel **A**: ALSO in 345 participants. Panel **B**: BLSO in 184 participants. Bar color represents scores of 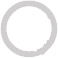
1 terrified, 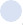
2 scared, 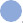
3 coping or 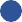
4 comfortable.

Complete data was available for 97% (n=345/355) of ALSO participants for pre- and immediate post-course paired testing of perceived confidence. For all items there was a strong and significant signal for an increase of median perceived confidence, with p<0.001 for all comparisons (median scores not shown). The proportion of participants who were terrified, scared, coping or comfortable were summarized and breech, vacuum, shoulder dystocia and neonatal resuscitation showed the greatest improvement in the proportion ‘comfortable’ from baseline ( Figure 3, Panel A).

Of 36 participants from the first ALSO cohort in 2008, 32 had a 12-month post-course evaluation: perceived confidence remained significantly higher than baseline for 8 of 13 situations; and was significantly lower for 5 of 13 situations at 12 months compared to the immediate post ALSO course evaluation ( Table 4).

**Table 4.** Perceived confidence of the first ALSO cohort on the Thailand-Myanmar border at baseline, immediately post course and at 12 months post baseline (n=32).

Obstetric Situation	Pre		Immediate post			12 Months Post			
	Median	IQR	Median	IQR	P-value ^a^	Median	IQR	P-value m12 *vs* baseline ^b^	P-value m12 vs immediate post ^c^
Bleeding in first trimester	3	[3-4]	4	[3-4]	**0.001**	3	[3-4]	0.059	0.275
Pre-eclampsia/ Eclampsia	2	[2-3]	3	[3-3]	**<0.001**	3	[2-4]	**0.005**	0.670
Bleeding in 3rd trimester	2	[2-3]	3	[3-4]	**<0.001**	3	[3-3]	**<0.001**	0.433
Preterm labour and PROM	3	[3-4]	3	[3-4]	0.068	4	[3-4]	0.073	1.000
Dysfunctional labour	2	[2-3]	3	[3-3]	**<0.001**	3	[2-3]	**0.002**	0.251
Malpresentation / Malposition/ Multiple	2	[2-3]	3	[2-3]	**<0.001**	3	[2-3]	**0.001**	0.577
Breech	3	[2-3]	3	[3-4]	**0.005**	3	[3-3]	0.060	0.059
Forceps	2	[1-2]	3	[2-3]	**<0.001**	2	[1-3]	0.062	**0.006**
Vacuum	3	[2-3]	4	[3-4]	**<0.001**	3	[2-3]	**0.009**	**0.007**
Shoulder Dystocia	3	[2-3]	3	[3-4]	**<0.001**	3	[2-3]	**0.003**	**0.010**
Post-partum Haemorrhage	3	[2-3]	3	[3-4]	**0.003**	3	[3-4]	**0.003**	0.582
Maternal resuscitation and trauma	2	[1-3]	3	[3-3]	**<0.001**	3	[2-3]	0.123	**0.015**
Neonatal resuscitation	3	[2-3]	4	[3-4]	**<0.001**	3	[3-4]	**0.007**	**0.027**

Scores 1 terrified 2 scared 3 coping 4 comfortable ^a^ Wilcoxon Signed Ranks Test paired data Immediate post compared to baseline ^b^ Wilcoxon Signed Ranks Test paired data 12 month post compared to baseline ^c^ Wilcoxon Signed Ranks Test paired data 12 month post compared to Immediate post

There were 89.3% (184/206) of participants on BLSO who completed pre- and immediate post-course evaluation. BLSO perceived confidence at baseline was low except on universal precautions; and the median post evaluation scores were significantly higher for all items (data not shown) p<0.001. The proportion of participants who were terrified, scared, coping or comfortable were summarized ( Figure 3, Panel B). Despite improvements most were still ‘scared’ of normal delivery at the end of the course.

### Thematic analysis course strengths

Almost all (97.7%; n=347/355) ALSO participants responded in their language of choice to the survey “What are the strengths of the course?”: Karen (21%), Burmese (16%) and English (63%). The most common themes to emerge from the responses were: “Improved knowledge”, “I can do it” and “Teamwork”.

***Improved Knowledge.*** Improved knowledge emerged as the strongest theme with nearly half the respondents including a statement on knowledge:

“I’m so happy to attend this ALSO training. I have learned many things I haven’t heard of before and improved my knowledge.” [midwife, migrant site, 2009],

“I know how to protect mothers from mortality and so we can reduce mother mortality. Before the course we never knew or heard of these things for the mother. Now we know.” [midwife, rural Myanmar, 2020].

“I got more knowledge on this ALSO course. The teaching was systematic for theory and practice so I can understand and appreciate it. I am happy and want to say thank you [midwife, refugee camp, 2008]

***I can do it.*** The words “I can…” in relation to the skills taught on ALSO were also frequently mentioned in the responses. Participants transmitted a strong sense of being empowered to solve obstetric problems because of the skills:

“I can do the skill. I cannot do it before the course. I am not afraid whereas before I was afraid.” [midwife, Mae Tao Clinic, 2008]

“I can apply and use the ALSO material on the patient. I have much less fear and more courage because I know how to handle the problem step by step.” [medic, migrant clinic, 2008]

“I learned more than just about safe delivery. I am just one person, but I think I can save the life of Karen women and children by this course. I feel useful.” [midwife, refugee camp, 2008]

***Teamwork.*** Participants also provided insights into the relationship of their skills and knowledge and their role, or sense of control in the team:

“With this training I have increased time awareness for emergencies. It helped me to have better teamwork because I can see how we organize and manage our team in the emergency.” [medic, migrant site, 2013]

“ALSO improved my skills in working together in a team and at the same time the importance of communication for the patient.” [midwife, refugee camp, 2009]

“I know what I am doing. I can help the team now.” [student midwife, refugee camp, 2010]

**Figure 4.**
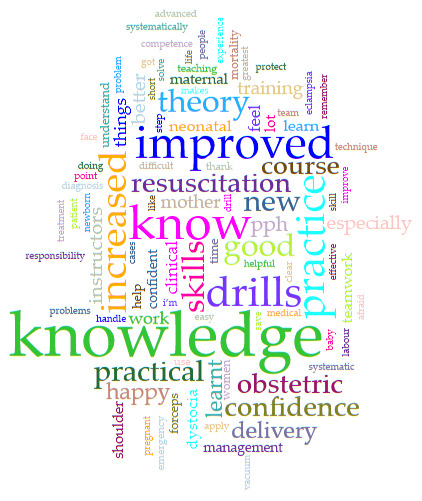
Word cloud resulting from analysis of all 347 full text comments of “What are the strengths of the course?”.

A summary of the most common terms from all the comments on the strength of the course from all participants were collated in a word cloud ( Figure 4).

### PPH related maternal mortality and stillbirth

There were 39,900 total births from 2000-2016 (excluding 2008) and there were 15 PPH related maternal deaths (PPH-related maternal mortality ratio (MMR) per 100,000 livebirths: 38.1 (95% CI 23.1-62.8), (15/39417). In the eight-year block preceding the implementation of ALSO and BLSO (2000-07) the PPH-related MMR was 57.0 (95% CI 30.06-108.3) (9/15797) and in eight years after (2009-16) was 25.4 (95% CI 11.6-55.4) (6/23620), p=0.109. There were 483 stillbirths, a stillbirth rate per 1000 total births of 12.1 (95% CI 11.1-13.2), with 18.2 (95% CI 16.2-20.4) (291/16016) before implementation and 11.1 (95% CI 9.8-12.5) (264/23884), p=0.038, after.

The proportion of PPH related mortality and stillbirths with associated factors were summarized ( Table 5). After adjustment, birth before ALSO and BLSO was implemented (aOR 3.825, 95% CI 1.1233-11.870), having migrant (not refugee) status (aOR 3.814, 95% CI 1.241-11.718) and attending four (or less) consultations (aOR 3.648, 95% CI 1.189-11.191) were all associated with PPH related mortality. Seven of the eight factors including birth before ALSO and BLSO was implemented (aOR 1.235, 95% CI 1.018-1.500) were associated with increased odds of stillbirth ( Table 5).

**Table 5.** Odds of PPH related maternal mortality and stillbirth before (2000-07) and after (2009–2016) the implementation of ALSO.

		% (n) PPH maternal mortality	Odds Ratio (95%CI)	P value	Adjusted Odds Ratio (95%CI)	P value	% (n) Stillbirth	Odds Ratio (95%CI)	P value	Adjusted Odds Ratio (95%CI)	P value
Before ALSO implemented	No	0.025 (6/23884)	Referent		Referent		1.1 (219/23884)	Referent		Referent	
	Yes	0.056 (9/16016)	2.24 (0.80- 6.29)	0.127	2.95 (1.01- 8.63)	0.015	1.4 (219/16016)	1.24 (1.04-1.49)	0.019	1.24 (1.02- 1.50)	0.033
Migrant	No	0.022 (5/23174)	Referent		Referent		1.2 (287/23174)	Referent		Not included	
	Yes	0.060 (10/16726)	2.77 (0.95- 8.11)	0.063	3.58 (1.19- 10.81)	0.021	1.2 (196/16726)	0.95 (0.79-1.14)	0.548		
Parity >4 (grand multiparity)	No	0.077 (12/36017)	Referent		Not included		1.1 (387/36017)	Referent		Referent	
	Yes	0.033 (3/3883)	2.32 (0.65- 8.22)	0.192			2.5 (95/3883)	2.33 (1.86-2.93)	<0.001	1.985 (1.56- 2.52)	<0.001
Smoking in pregnancy	No	0.055 (10/30647)	Referent		Not included		1.0 (321/30647)	Referent		Referent	
	Yes	0.033 (5/9123)	1.68 (0.57- 4.92)	0.344			1.8 (160/9123)	1.69 (1.39-2.04)	<0.001	1.27 (1.03- 1.56)	0.025
Underweight (<40 kg)	No	0.037 (14/37621)	Referent		Not included		1.2 (440/37621)	Referent		Referent	
	Yes	0.045 (1/2212)	1.22 (0.16- 9.24)	0.851			1.7 (38/2212)	1.48 (1.06-2.06)	0.022	1.59 (1.13- 2.25)	0.007
Antenatal consultations ≤4	No	0.029 (10/34291)	Referent		Referent		1.0 (344/34291)	Referent		Referent	
	Yes	0.089 (5/5609)	3.06 (1.05- 8.95)	0.041	3.78 (1.26- 11.37)	0.018	2.5 (139/5609)	2.51 (2.06-3.06)	<0.001	2.53 (2.05- 3.12)	<0.001
Anaemic first ANC visit (HCT <30%)	No	0.034 (12/35242)	Referent		Not included		1.1 (380/35242)	Referent		Referent	
	Yes	0.068 (3/4382)	2.19 (0.57- 7.13)	0.279			2.3 (101/4382)	2.16 (1.73-2.70)	<0.001	1.69 (1.34- 2.14)	<0.001
Falciparum malaria in pregnancy	No	0.037 (14/37444)	Referent		Not included		1.2 (434/37444)	Referent		Referent	
	Yes	0.041 (1/2455)	1.09 (0.14- 8.29)	0.934			2.0 (49/2455)	1.74 (1.29-2.34)	<0.001	1.45 (1.06- 1.98)	0.020

Missing data (n): includes parity (1), underweight (67), anaemia (278), falciparum (1) Abbreviations: CI=confidence interval; ALSO=Advanced Life Support in Obstetrics; ANC=antenatal care; HCT=haematocrit; PPH= post-partum haemorrhage.

## Discussion

This evaluation of ALSO and BLSO as education tools for health care workers mostly without tertiary qualifications in a post-conflict RLS demonstrates that they can achieve excellent standards for practical components of emergency care in obstetrics. The increased perceived confidence on most items, including at 12 months post baseline, and self-declared benefits from the training in knowledge, skills and teamwork, is consistent with former evaluations in other settings ^6– 8^. The risk of PPH-related maternal mortality and stillbirth declined following the implementation of ALSO and BLSO in this setting, suggesting a meaningful impact on the quality of care.

On the Thailand-Myanmar border, an area of protracted conflict resulting in disrupted education systems, the written theory test has proved to be challenging for health staff ^29^. Approximately one in six participants attended (not necessarily completed) tertiary education in Myanmar though none studied health related subjects. Despite low overall pass marks on theory results ‘increased knowledge’ emerged as one the strongest themes from the qualitative analysis. However, unlike the ALSO course administration in high income countries, this increased knowledge required months of guided study. Provision of the syllabus without facilitation was impossible given the baseline education and diverse language abilities among the staff.

BLSO on the Thailand-Myanmar border has been used as a confidence builder and tool for raising awareness in maternal and child health for staff working mostly outside the delivery room. While BLSO significantly increased perceived confidence, the short nature of this course is no replacement for comprehensive midwifery and nursing training. This is perhaps best reflected by the participants’ self-assessment, where 50% reported being ‘scared’ of normal vaginal delivery after the BLSO course.

The use of historic comparison, before and after ALSO and BLSO were implemented, is a weakness of this evaluation, as changes in the time frame may have occurred that impact on PPH related maternal mortality and stillbirth, and have not been accounted. Nevertheless, the factors associated with PPH maternal mortality highlight known problems in the area related to access to antenatal care ^30– 32^, and delivery before or after implementation of the courses remained a significant factor after adjustment for known confounders. Reduction of MMR over time is not smooth or inevitable in this setting. The PPH-related MMR (25.4 per 100,000 live births) on the Thailand Myanmar border after ALSO and BLSO were implemented was higher than the all cause official maternal mortality ratio in Thailand ^33^. Changes were unrelated to availability of uteronics - syntocinon, methergyn, and misoprostol have been available since before 2000 and tranexamic acid was not introduced until 2017. While researchers could not retrospectively assess if the team-based approach to management of PPH taught in ALSO and BLSO training used for women experiencing PPH in SMRU delivery rooms after 2008, the qualitative and quantitative results presented here suggest that the methods learned were implemented.

The factors significantly associated with stillbirth including grand multiparity, smoking, underweight, four or less antenatal consultations, anaemia and falciparum malaria, all typical of RLS also stress the need for improved antenatal care and may in part be addressed by improved access to family planning ^34^. SMRU implemented the WHO partogram in 1994 and while the stillbirth rate of 12.1 (95%CI 11.1-31.2) per 1000 total births is at the global target, like maternal mortality it may well be under-estimated ^35^.

ALSO sets a standard. In protracted conflict and post-conflict settings where education as well as health are neglected reaching standard levels of practice are critical ^29, 36^. Standards are the main reasons for the minimal modifications of ALSO on the Thailand-Myanmar border, limited to only those situations that are extremely rare or skills requiring equipment that is unavailable. This long and positive evaluation with improved outcomes while ALSO and BLSO have been the only emergency obstetric courses on offer in SMRU clinics and where the level 4 data was reviewed, supports their benefit ^37– 39^ and sustainability in RLS ^40^.

### Limitations

This is an evaluation of a single emergency obstetric course, although many exist, and the comparison for PPH related maternal mortality and stillbirth is historical which is acknowledged as imperfect ^41^. Deeper analysis into the factors associated with each case of PPH related mortality e.g. timeliness of uterotonics; and stillbirth e.g. pre or intrapartum; would also be helpful to understand if ALSO skills could have prevented the adverse outcome ^42^. ALSO relies on mannequins for the practical component and is a burden for most RLS to purchase or maintain but invaluable in visualization of emergency obstetric skills. A complete cost analysis has not been conducted but the cost of replacing mannequins far exceeds other facility-based course costs as instructors work on a voluntary basis ^43^.

## Conclusion

The best obstetric emergency course for RLS is unknown, however ALSO and BLSO are clearly feasible, effective and sustainable for adult education.

## Data availability

### Underlying data

Oxford University Research Archive: ALSO and BLSO evaluation Thailand Myanmar border. https://doi.org/10.5287/bodleian:ErmK5jpO7 ^26^.

The project contains the following source data:

- ALSO Eval upload (ALSO raw data)
- BLSO Eval upload (BLSO raw data)

PPHMD_SBuploadv2 (Data for the quantitative analysis of maternal death from post-partum haemorrhage and stillbirths pre and post ALSO (8 year blocks))

### Extended data

Oxford University Research Archive: ALSO and BLSO evaluation Thailand Myanmar border. https://doi.org/10.5287/bodleian:ErmK5jpO7 ^26^.

The project contains the following extended data:

- ALSO.docx (ALSO evaluation tool)
- BLSO.docx (BLSO evaluation tool)

Data are available under the terms of the Creative Commons Attribution 4.0 International license (CC-BY 4.0).
