# Supplementary material for: A mixed methods evaluation of Advanced Life Support in Obstetrics (ALSO) and Basic Life Support in Obstetrics (BLSO) in a resource-limited setting on the Thailand-Myanmar border
Source: Wellcome Open Res. 2021 Jun 28;6:94. Originally published 2021 May 5. [Version 2] doi: 10.12688/wellcomeopenres.16599.2 (PMC8204190; doi:10.12688/wellcomeopenres.16599.2)
Supplement: Peer review history

# Peer review: Reviewer response for version 2

**DOI:** 10.21956/wellcomeopenres.18767.r44677

**Published:** 2021-06-30

**Authors:** Shahla Y. Namak

**Affiliations:** 1 Department of Family and Community Medicine, Wake Forest School of Medicine, Winston Salem, NC, USA; 2 American Academy of Family Medicine, Society of Teachers of Family Medicine, Leawood, KS, USA

I reviewed the updates edits and seems good. No further comments

Is the work clearly and accurately presented and does it cite the current literature?

Yes

If applicable, is the statistical analysis and its interpretation appropriate?

I cannot comment. A qualified statistician is required.

Are all the source data underlying the results available to ensure full reproducibility?

No source data required

Is the study design appropriate and is the work technically sound?

Yes

Are the conclusions drawn adequately supported by the results?

Yes

Are sufficient details of methods and analysis provided to allow replication by others?

Partly

Reviewer Expertise:

NA

I confirm that I have read this submission and believe that I have an appropriate level of expertise to confirm that it is of an acceptable scientific standard.

# Peer review: Reviewer response for version 1

**DOI:** 10.21956/wellcomeopenres.18295.r44112

**Published:** 2021-06-14

**Authors:** Lee T Dresang

**Affiliations:** 1 Department of Family Medicine and Community Health, University of Wisconsin, Madison, WI, USA

Thank you for this valuable addition to research documenting the value of the Advanced Life Support in Obstetrics (ALSO) and Basic Life Support in Obstetrics (BLSO) courses. While previous studies have documented increased confidence level with the ALSO/BLSO course, this is the first study to do so in an area of protracted conflict. 

 The model for studying the syllabus as a group over time is creative and effective. It may be a helpful model in other resource limited settings. 

 The analysis in the context of Kirkpatrick's framework is helpful. The level 4 data on the course's impact of maternal mortality ratio (MMR) and stillbirth rates is especially interesting. While the MMR decreased from 57.0 to 25.4 pre to post-ALSO/BLSO, the change is not statistically significant since the confidence intervals overlap (30.06-108.3) and (23.1-62.8), respectively. The decrease in stillbirth rate was statistically significant (16.2-20.4) vs (9.8-12.5).

 As the authors acknowledge, the study design (pre-post analysis) has limitations. Changes noted may be due to factors other than the ALSO/BLSO course. A future study with a control group could better analyze the impact of ALSO/BLSO alone. 

 For the Odds Ratio, I believe fewer digits would be appropriate. Could report to 100 ^th ^(2 decimal places) rather than 10,000 ^th^(4 decimal places).

 Page 4, last paragraph:

 Effect of malaria interesting

 Great to have in Karen, Burmese and English.  

 Page 6, table 1:

 Normal labor taught in BLSO; not in ALSO – would leave ALSO column blank on the row containing Normal labor in BLSO column.

 ALSO teaches “Intrapartum fetal surveillance” BLSO has basics in “Emergent/urgent prenatal Assessment” chapter with optional “electronic fetal monitoring” section for medical students in the “Normal labor” chapter.

 BLSO also teaches Pre-eclampsia/Eclampsia

 BLSO chapter is titled “Breech, Twin Gestation, Cord Prolapse, and Other Malpresentations”; Covers these topics, just in a more basic manner than in ALSO. Prehospital personnel may need to attend emergent breech delivery. ALSO main focus is also on emergent (not planned) vaginal birth

 Neonatal resuscitation is not included in the 9 ^th^ edition of ALSO; Most providers in the US take the neonatal resuscitation program course instead; Neonatal resuscitation is still in the BLSO course

 ALSO starts its course with Safety in Maternity Care; BLSO does not have safety in maternity care chapter; it has a short section in normal labor chapter recommending universal precautions

 9 ^th^ edition of ALSO has a Maternal Sepsis chapter but there is little material on Malaria. There is a full chapter on Malaria in Global ALSO Manual.

 Page 6 “ALSO for the Thailand-Myanmar border”

 Adaptations are appropriate; the Global ALSO manual is available to help with adapting ALSO courses to local circumstances, especially in resource limited settings

 Nice creative model of reviewing ALSO chapters over time in preparation for in person course especially given language and other barriers in study setting.

 Page 10 Love the comments and the word cloud!

 Page 10 last paragraph

 Would cut “(95% CI)” on the 4 ^th^ line of the paragraph and add “95%” before “CI 23.1-62.8)”

 Would add studies by Sorensen:

 ​​​​​​Sorensen B, Rasch V, Massawe S, Nyakina J, Elsass P, Nielsen B. Impact of ALSO® training on the management of prolonged labor and neonatal care at Kagera Regional Hospital, Tanzania. *Int J Gynaecol Obstet. *2010;111(1):8-12. ^1^

 Sorensen B, Rasch V, Massawe S, Nyakina J, Elsass P, Nielsen B. Advanced Life Support in Obstetrics (ALSO®) and post-partum hemorrhage: a prospective intervention study in Tanzania. *Acta Obstet Gynecol Scand. *2011;90(6):609-614. ^2^

 And Bower:

 ​​​​​​Bower D, Wolkomir M, Schubot D. The effects of the ALSO® course as an educational intervention for residents. *Family Medicine. *1997;29(3):187-193. ^3^

 Thank you again for this important, well written article!

Is the work clearly and accurately presented and does it cite the current literature?

Yes

If applicable, is the statistical analysis and its interpretation appropriate?

Yes

Are all the source data underlying the results available to ensure full reproducibility?

Yes

Is the study design appropriate and is the work technically sound?

Yes

Are the conclusions drawn adequately supported by the results?

Yes

Are sufficient details of methods and analysis provided to allow replication by others?

Yes

Reviewer Expertise:

Family Medicine Obstetrics

I confirm that I have read this submission and believe that I have an appropriate level of expertise to confirm that it is of an acceptable scientific standard.

## References

[1] : Impact of ALSO training on the management of prolonged labor and neonatal care at Kagera Regional Hospital, Tanzania. *Int J Gynaecol Obstet* .2010;111(1) : 10.1016/j.ijgo.2010.04.031 8-12 10.1016/j.ijgo.2010.04.031 20646704

[2] : Advanced life support in obstetrics (ALSO) and post-partum hemorrhage: a prospective intervention study in Tanzania. *Acta Obstet Gynecol Scand* .2011;90(6) : 10.1111/j.1600-0412.2011.01115.x 609-14 10.1111/j.1600-0412.2011.01115.x 21388368

[3] : The effects of the ALSO course as an educational intervention for residents. Advanced Life Support in Obstetrics. *Fam Med* .1997;29(3) :187-93 9085101

# Response

**Published:** 2021-06-18

**Authors:** Rose McGready

**Affiliations:** Shoklo Malaria Research Unit, Thailand

Thank you for this valuable addition to research documenting the value of the Advanced Life Support in Obstetrics (ALSO) and Basic Life Support in Obstetrics (BLSO) courses. While previous studies have documented increased confidence level with the ALSO/BLSO course, this is the first study to do so in an area of protracted conflict. 

 The model for studying the syllabus as a group over time is creative and effective. It may be a helpful model in other resource limited settings. 

 The analysis in the context of Kirkpatrick's framework is helpful. The level 4 data on the course's impact of maternal mortality ratio (MMR) and stillbirth rates is especially interesting. While the MMR decreased from 57.0 to 25.4 pre to post-ALSO/BLSO, the change is not statistically significant since the confidence intervals overlap (30.06-108.3) and (23.1-62.8), respectively. The decrease in stillbirth rate was statistically significant (16.2-20.4) vs (9.8-12.5).

 As the authors acknowledge, the study design (pre-post analysis) has limitations. Changes noted may be due to factors other than the ALSO/BLSO course. A future study with a control group could better analyze the impact of ALSO/BLSO alone. 

**For the Odds Ratio, I believe fewer digits would be appropriate. Could report to 100** **^th^** **(2 decimal places) rather than 10,000** **^th^** **(4 decimal places).**

*Amended*

 Page 4, last paragraph:

 Effect of malaria interesting

*Thank you for noticing (most wouldn’t). This is of great concern locally given the current deterioration in the health system in Myanmar.*

 Great to have in Karen, Burmese and English.  

*Most of the staff still prefer to have two languages available to read during the exam time and Karen, which does not have the same broad vocabulary as Burmese and English, takes more printed pages but these approaches have not been systematically documented.*

 Page 6, table 1: *Thank you for being prescriptive here for corrections* *to this table.*

 Normal labor taught in BLSO; not in ALSO – would leave ALSO column blank on the row containing Normal labor in BLSO column.

*Agree. Amended*

 ALSO teaches “Intrapartum fetal surveillance” BLSO has basics in “Emergent/urgent prenatal Assessment” chapter with optional “electronic fetal monitoring” section for medical students in the “Normal labor” chapter.

*Electronic fetal monitoring is not available. The table is amended so that this perspective is clarified.*

 BLSO also teaches Pre-eclampsia/Eclampsia

*Amended*

 BLSO chapter is titled “Breech, Twin Gestation, Cord Prolapse, and Other Malpresentations”; Covers these topics, just in a more basic manner than in ALSO. Prehospital personnel may need to attend emergent breech delivery. ALSO main focus is also on emergent (not planned) vaginal birth

*Amended*

 Neonatal resuscitation is not included in the 9 ^th^ edition of ALSO; Most providers in the US take the neonatal resuscitation program course instead; Neonatal resuscitation is still in the BLSO course

*Neonatal resuscitation has been retained. There are no alternative courses in the area; and we have noted this by a new footnote to table 1.*

*As an aside, we have published previously on this topic as no assisted ventilation is possible at the field sites (see: doi: 10.1371/journal.pone.0190419 and ALSO ® is mentioned in the methods section).*

 ALSO starts its course with Safety in Maternity Care; BLSO does not have safety in maternity care chapter; it has a short section in normal labor chapter recommending universal precautions

*Agree, amended – table order amended.*

 9 ^th^ edition of ALSO has a Maternal Sepsis chapter but there is little material on Malaria. There is a full chapter on Malaria in Global ALSO Manual.

*Differentiating malaria and sepsis and eclampsia is important. The management and especially the drugs are different. In low transmission settings such as the Thailand-Myanmar border, there is no protective immunity and pregnant women are at increased risk of mortality. A pregnant woman with convulsions due to P. falciparum can mostly be managed by appropriate drug treatment (+/- blood transfusion) without the need to deliver; unlike eclampsia where the role of urgent delivery is high on the management agenda. Time is spent during a case untangling eclampsia, sepsis and malaria, all of which can increase maternal and fetal mortality and morbidity. The trade-off to spending extra time on diseases that are important causes of morbidity and mortality in the local setting was less time spent on causes of maternal morbidity and mortality that cannot be readily diagnosed or treated in this setting, or contribute to a small percentage of maternal deaths compared to high income settings (e.g. thromboembolic disease).*

*See the next response too please.*

 Page 6 “ALSO for the Thailand-Myanmar border”

 Adaptations are appropriate; the Global ALSO manual is available to help with adapting ALSO courses to local circumstances, especially in resource limited settings

*Thank you. A comment is now included in the methods in relation to this and the above comment and the reference to Rojas-Suarez et al., 2017 (as below).*

 Nice creative model of reviewing ALSO chapters over time in preparation for in person course especially given language and other barriers in study setting.

*Thank you.*

 Page 10 Love the comments and the word cloud!

*Thank you – it expresses a different view of what low resource setting participants value.* *We were afraid it would not get well reviewed.*

 Page 10 last paragraph

 Would cut “(95% CI)” on the 4 ^th^ line of the paragraph and add “95%” before “CI 23.1-62.8)”

*Amended.*

 Would add studies by Sorensen:

 ​​​​​​Sorensen B, Rasch V, Massawe S, Nyakina J, Elsass P, Nielsen B. Impact of ALSO® training on the management of prolonged labor and neonatal care at Kagera Regional Hospital, Tanzania. *Int J Gynaecol Obstet. *2010;111(1):8-12. ^1^

 Sorensen B, Rasch V, Massawe S, Nyakina J, Elsass P, Nielsen B. Advanced Life Support in Obstetrics (ALSO®) and post-partum hemorrhage: a prospective intervention study in Tanzania. *Acta Obstet Gynecol Scand. *2011;90(6):609-614. ^2^

 And Bower:

 ​​​​​​Bower D, Wolkomir M, Schubot D. The effects of the ALSO® course as an educational intervention for residents. *Family Medicine. *1997;29(3):187-193. ^3^

 These relevant references have been included. I hope the editor remains agreeable as the journal recommends a maximum of 40: we are now at 43.

 Thank you again for this important, well written article!

- **Is the work clearly and accurately presented and does it cite the current literature?**

Yes

- **Is the study design appropriate and is the work technically sound?**

Yes

- **Are sufficient details of methods and analysis provided to allow replication by others?**

Yes

- **If applicable, is the statistical analysis and its interpretation appropriate?**

Yes

- **Are all the source data underlying the results available to ensure full reproducibility?**

Yes

- **Are the conclusions drawn adequately supported by the results?**

Yes

**References**

 1. Sorensen BL, Rasch V, Massawe S, Nyakina J, et al.: Impact of ALSO training on the management of prolonged labor and neonatal care at Kagera Regional Hospital, Tanzania. *Int J Gynaecol Obstet*. 2010; **111** (1): 8-12 PubMed Abstract | Publisher Full Text

 2. Sorensen BL, Rasch V, Massawe S, Nyakina J, et al.: Advanced life support in obstetrics (ALSO) and post-partum hemorrhage: a prospective intervention study in Tanzania. *Acta Obstet Gynecol Scand*. 2011; **90** (6): 609-14 PubMed Abstract | Publisher Full Text

 3. Bower DJ, Wolkomir MS, Schubot DB: The effects of the ALSO course as an educational intervention for residents. Advanced Life Support in Obstetrics. *Fam Med*. 1997; **29** (3): 187-93 PubMed Abstract

 Competing Interests

*No competing interests were disclosed.*

 Reviewer Expertise

 Family Medicine Obstetrics

**I confirm that I have read this submission and believe that I have an appropriate level of expertise to confirm that it is of an acceptable scientific standard.**

*We appreciate the time the reviewer has taken to improve the quality of this manuscript. The suggestions were constructive and supportive.*

# Peer review: Reviewer response for version 1

**DOI:** 10.21956/wellcomeopenres.18295.r44114

**Published:** 2021-06-07

**Authors:** Shahla Y. Namak

**Affiliations:** 1 Department of Family and Community Medicine, Wake Forest School of Medicine, Winston Salem, NC, USA; 2 American Academy of Family Medicine, Society of Teachers of Family Medicine, Leawood, KS, USA

I believe it is a very good study and report to be published.

 The article addresses improved confidence in clinical performance hands on skills and knowledge after receiving training in ALSO and BLSO Obstetrics Emergency Courses. In a post-conflict limited resource setting Thailand-Myanmar border. Other outcomes studied were the rates of MMR and stillbirth comparing the intervals pre-course from 2000-2007 and post-course 2008-2020.

 Methods including participants, who were eligible for the ALSO and BLSO courses, the structure of the courses were reported and the assessment of the educational effectiveness qualitative, quantitative then chart review.

 It is unclear in the methods page 6/16, the first paragraph under Table 1 the ALSO course was standard 2 day course but the mentioned the preparation phase reviewed 1-2 chapters per week (60-120) minutes. (Question: How many days/weeks took for the preparation of the chapters) the ALSO course is designed as a 2 day course.

 How many questions were used for the ALSO written examination? you mentioned BLSO using 10 questions.

 Results: Results were clear in regard to passing rates for both ALSO and BLSO. the clinical assessment hands on were high rate passing >90%, but the written exam was 50% passing for ALSO evaluation. the reported self assessment of the participants was showing clear confidence in 8/13  situations which seems to persist after 12 months.

 The comparison of the outcome related to PPH caused mortally pre-course and post-course were positive results. although I agree with the limitation mentioned here that they did not look at the reasons/ etiology/risk factors for PPH if preventable or not, the availability of uterotonic in a resource limited area. And I think another limitation included: researchers did not have the chance to study if the participants in these PPH scenarios had received the ALSO course training? Did they implement what they learned and used a team based approach?

 The stillbirth result was reported well with a good conclusion.

 The tables numbering are incorrect:

- Table 3 is mentioned twice
- Page 12/16, table 3 needs to change to table 4- correct link to this table on page 8/16
- Table 4 on page 13/16 needs to change to Table 5 and the link to this tale needs to be corrected on page 11/16.

Thank you for allowing me to review this review. I enjoyed reading it.

 Shahla Namak, MD Associate Professor, Family and community Medicine at Wake Forest School of Medicine.

Is the work clearly and accurately presented and does it cite the current literature?

Yes

If applicable, is the statistical analysis and its interpretation appropriate?

I cannot comment. A qualified statistician is required.

Are all the source data underlying the results available to ensure full reproducibility?

No source data required

Is the study design appropriate and is the work technically sound?

Yes

Are the conclusions drawn adequately supported by the results?

Yes

Are sufficient details of methods and analysis provided to allow replication by others?

Partly

Reviewer Expertise:

Maternal health, global health, as an American Academy of Family Medicine and an Advanced life support of Obstetrics instructor I teach and published about this course in a LMIC. Refugee health and challenges.

I confirm that I have read this submission and believe that I have an appropriate level of expertise to confirm that it is of an acceptable scientific standard.

# Response

**Published:** 2021-06-18

**Authors:** Rose McGready

**Affiliations:** Shoklo Malaria Research Unit, Thailand

Reviewer 1

 I believe it is a very good study and report to be published.

 The article addresses improved confidence in clinical performance hands on skills and knowledge after receiving training in ALSO and BLSO Obstetrics Emergency Courses. In a post-conflict limited resource setting Thailand-Myanmar border. Other outcomes studied were the rates of MMR and stillbirth comparing the intervals pre-course from 2000-2007 and post-course 2008-2020.

 Methods including participants, who were eligible for the ALSO and BLSO courses, the structure of the courses were reported and the assessment of the educational effectiveness qualitative, quantitative then chart review.

*Thank you*

 It is unclear in the methods page 6/16, the first paragraph under Table 1 the ALSO course was standard 2 day course but the mentioned the preparation phase reviewed 1-2 chapters per week (60-120) minutes. (Question: How many days/weeks took for the preparation of the chapters) the ALSO course is designed as a 2 day course.

*Thank you. It is correct that it is designed as a 2 day course and we run the 2 day course. However due language and education typical of the border area we also spend a pre-course preparation phase predominantly in support of the theory component which is explained in the next paragraph, under table 1, cut and pasted here for clarity:*

*“ALSO course teaching was structured into two parts: the preparation phase and the standard two-day intensive course. The preparation phase reviewed one to two chapters per week, mostly in English (60-120 minutes, led by a doctor). A study group for 2-3 hours later in the week, mostly in Karen and Burmese language led by a senior midwife or medic included practice of skills using donated mannequins. The total preparation phase took 12-16 weeks.”*

 How many questions were used for the ALSO written examination? you mentioned BLSO using 10 questions.

*We do 50 – there are some questions e.g. on electronic fetal surveillance that are not applicable in this setting. The sentence has been amended as follows: “*The ALSO written exam was also modified for relevance and clarity, removing difficult English e.g. double negatives, and electronic fetal surveillance, leaving 50 questions”.

 Results: Results were clear in regard to passing rates for both ALSO and BLSO. the clinical assessment hands on were high rate passing >90%, but the written exam was 50% passing for ALSO evaluation. the reported self assessment of the participants was showing clear confidence in 8/13  situations which seems to persist after 12 months.

 The comparison of the outcome related to PPH caused mortally pre-course and post-course were positive results. although I agree with the limitation mentioned here that they did not look at the reasons/ etiology/risk factors for PPH if preventable or not, the availability of uterotonic in a resource limited area.

*The uterotonics available in this area at the time of this evaluation have been added in the discussion section for clarity: *Changes were unrelated to availability of uteronics - syntocinon, methergyn, and misoprostol have been available since before 2000 and tranexamic acid was not introduced until 2017.

 And I think another limitation included: researchers did not have the chance to study if the participants in these PPH scenarios had received the ALSO course training? Did they implement what they learned and used a team based approach?

*This important point has been included in the discussion.*

 The stillbirth result was reported well with a good conclusion.

*Thank you*

 The tables numbering are incorrect:  Table 3 is mentioned twice

**Page 12/16, table 3 needs to change to table 4- correct link to this table on page 8/16

  Table 4 on page 13/16 needs to change to Table 5 and the link to this tale needs to be corrected on page 11/16. *Thank you for pointing this out. It is our error and has now been amended.*

 Thank you for allowing me to review this review. I enjoyed reading it.

 Shahla Namak, MD Associate Professor, Family and community Medicine at Wake Forest School of Medicine.

- **Is the work clearly and accurately presented and does it cite the current literature?**

Yes

- **Is the study design appropriate and is the work technically sound?**

Yes

- **Are sufficient details of methods and analysis provided to allow replication by others?**

Partly

- **If applicable, is the statistical analysis and its interpretation appropriate?**

I cannot comment. A qualified statistician is required.

- **Are all the source data underlying the results available to ensure full reproducibility?**

No source data required

- **Are the conclusions drawn adequately supported by the results?**

Yes

 Competing Interests

*No competing interests were disclosed.*

 Reviewer Expertise

 Maternal health, global health, as an American Academy of Family Medicine and an Advanced life support of Obstetrics instructor I teach and published about this course in a LMIC. Refugee health and challenges.

**I confirm that I have read this submission and believe that I have an appropriate level of expertise to confirm that it is of an acceptable scientific standard.**

*We appreciate the time taken by the reviewer to read this manuscript in detail and provide constructive feedback.*
